# Editorial: Understanding plant responses and phytoremediation strategies for arsenic toxicity

**DOI:** 10.3389/fpls.2026.1866458

**Published:** 2026-06-10

**Authors:** Zahra Souri, Muhammad Ansar Farooq, Marek Vaculík

**Affiliations:** 1 Department of Biology, Faculty of Science, Razi University, Kermanshah, Iran; 2 Institute of Environmental Sciences and Engineering (IESE), School of Civil and Environmental Engineering (SCEE), National University of Sciences and Technology (NUST), Islamabad, Pakistan; 3 CFAES Rattan Lal Center for Carbon Management and Sequestration, School of Environment and Natural Resources, The Ohio State University, Columbus, OH, United States; 4 Department of Plant Physiology, Faculty of Natural Sciences, Comenius University in Bratislava, Bratislava, Slovakia; 5 Institute of Botany, Plant Science and Biodiversity Centre, Slovak Academy of Sciences, Bratislava, Slovakia

**Keywords:** arsenic toxicity, nano-biochar, non-invasive detection, phytoremediation, soil amendments, stress mitigation

## Non-invasive detection of As stress in plants – a challenge for future?

Rapid and accurate diagnosis of environmental stressors, such as arsenic (As), is essential for effective crop management, as As disrupts cellular function and threatens food security and ecosystem resilience (Souri et al., 2022; Patel et al., 2023; Sinha et al., 2023). Countering As toxicity in the field requires a multifaceted scientific approach, where novel non-invasive methods for assessing plant responses offer a major improvement over conventional destructive techniques. Juárez et al. reported correlation of specific spectral fingerprints with established biochemical markers linked to lignin and phenolic metabolism and achieved approximately 89% accuracy in detecting As using Raman spectroscopy. The study enabled early detection of As toxicity before the onset of visible symptoms, thus allowing real-time phenotyping of As tolerance. Another report by Choudhary et al. (2025) employed biospeckle optical coherence tomography (bOCT), a non-invasive imaging technique capable of monitoring dynamic cellular activity and suggested it as a reliable tool for detecting As-induced stress in lentil plants. Additionally, Capabianco et al. (2026) using hyperspectral imaging (HSI) in the short-wave infrared range, combined with micro-X-ray fluorescence (micro-XRF) and spectroradiometric measurements obtained with a portable device on *Pteris vittata* showed that innovative multi-analytical approach can develop efficient and scalable tools for optimizing As-phytoremediation strategies and environmental monitoring. This research offers an integrated strategy, from new diagnostic tools to engineered interventions, moving beyond single-method approaches.

## Mitigation of As toxicity by soil amendments and nano-based materials

Once precise diagnostics are established, the focus shifts to mitigating As toxicity in plants. Proposed strategies include amendments that leverage interactions with elements such as silicon (Si), sulfur (S), and selenium (Se) (Vaculík et al., 2020; Saifullah et al., 2018; Souri et al., 2021; Karimi et al., 2025), as well as the application of materials like biochar, nano-iron, and others (e.g., Batool et al., 2025; Iqbal et al., 2025; Su et al., 2025). From this perspective, two studies address the issue through material science, providing complementary “bottom-up” solutions. Fang et al. presented a sophisticated engineered composite termed “nano-ferro-silicon biochar” (NNFB), by embedding γ-Fe_2_O_3_ and SiO_2_ nanoparticles into rice straw biochar. This report suggested a synergistic soil amendment that tackles As contamination on multiple fronts: the porous biochar matrix sequestered toxins and improved soil health, the embedded nano-iron oxides specifically adsorbed and immobilized As, and the Si component enhanced the plant’s own physiological defenses. This “three-in-one” strategy simultaneously locked As in the soil, improved the soil matrix, fortified the plant stress tolerance, exemplifying the next generation of agronomic tools by translating fundamental knowledge of As chemistry and plant Si benefits into a single, applicable technology. Further experiments of Fang et al. demonstrated that NNFB effectively adsorbed arsenite [As(III)] from both solution and contaminated soil. In hydroponic and pot trials with rice, application of NNFB (0.25–1.0%) significantly alleviated As toxicity across multiple growth stages. The mechanism involved reducing reactive oxygen species (ROS) accumulation by lowering H_2_O_2_ content and enhancing peroxidase (POD) activity, alongside downregulating key As transporter genes (OsLsi1, OsLsi2, and OsABCC1) to decrease As uptake and translocation. Most notably, in soil contaminated with 40 mg/kg As(III), the addition of 0.5% and 1% NNFB dramatically increased grain yield, with the seed number per plant rising by nearly 7- and 8-fold, respectively, compared to the As-stressed control. Therefore, NNFB was suggested to be a promising multi-functional soil amendment for simultaneously remediating As contamination and enhancing crop productivity in affected agricultural systems (Fang et al.).

Li et al. provided a crucial, nuanced comparison when evaluated zinc (Zn), copper (Cu), and Si amendments in three applied forms as nanoparticles (NPs), ionic salts, and bulk particles (BPs) on growth of As treated rice, a staple food for over 3.5 billion people. Their work revealed that under As stress, Zn amendments increased grain yield by 4.6–7.3 times, with ionic ZnSO_4_ performing best by fully restoring yield to background levels and reducing the toxic As(III)/total As ratio in grains by 46.9%. Cu amendments also boosted yield by 3.8–5.6 times and significantly lowered the As(III)/total As ratio by 20.2–65.6%. Nanoparticles provide some relief from As toxicity but do not promote rice growth more effectively than ionic or bulk forms. Nano-agriculture should focus on scalable, safe amendments. Zinc and copper show promise for protecting crops under stress, but more field validation is needed.

## A novel insight to As phytoremediation and phytomanagement of As-contaminated sites

Key priorities are innovative soil As remediation and advanced phytomanagement tools. Modern technologies accelerate breeding of resilient crops and enable precise field monitoring, providing critical feedback to improve As mitigation strategies. Kama et al. offered an elegant, nature-inspired strategy of intercropping two crops with As-hyperaccumulating plants. The study designed a phytoremediation consortium where each plant played a complementary role: the hyperaccumulating fern (*Pteris cretica*) acting as the primary As extractor, the deep-rooted spinach (*Spinacia oleracea* L.) aided in soil structure and nutrient cycling, while the peanut (*Arachis hypogaea* L.), a valuable legume crop provided economic yield and nitrogen fixation. The pot experiment demonstrated that intercropping systems, particularly the three-species combination (*A. hypogaea*, *P. cretica*, *S. oleracea*), significantly enhanced soil As removal, achieving an 88% removal rate compared to monocropped peanuts. Concurrently, As uptake by peanut plants was substantially reduced, minimizing the risk of food chain contamination (Kama et al.). This system achieved what single-species phytoremediation often cannot: enhanced As removal, improved soil health, and economic viability for landowners. It shifted the paradigm from land removal to sustainable production, a crucial step for practical adoption by farming communities. Kama et al. further demonstrated that intercropping with hyperaccumulator plant diversity offers a sustainable and effective strategy for remediating As-contaminated agricultural land while ensuring food safety.

This research reports advances in non-invasive arsenic stress diagnostics, nanoremediation, and phytomanagement. However, gaps remain in long-term field performance, cost-effectiveness, and ecological risks. Future research should focus on multi-site field validation, portable real-time detection devices, and multi-omics integration to understand molecular mechanisms of arsenic tolerance and hyperaccumulation in plants.

## References

[B1] Batool A. Farooq M. A. Iqbal M. M. Ashraf U. Imran M. Abbas G. . (2025). Nitrogen-doped carbon quantum dots enhance rice seedlings growth and antioxidant defense system under arsenic stress. Ecotoxicology Environ. Saf. 307, 119413. doi: 10.1016/j.ecoenv.2025.119413 41240570

[B2] Capobianco G. Bonifazi G. Serranti S. Trotta O. Cucuzza P. Antenozio M. L. . (2026). Monitoring of arsenic uptake in Pteris vittata using short-wave infrared spectroscopy during multi-scale field trial. Spectrochim. Acta Part. A. Mol. Biomolecular Spectrosc. 347, 126964. doi: 10.1016/j.saa.2025.126964 40987179

[B3] Choudhary A. A. K. Devi A. Tyagi L. Rajagopalan U. M. Kadono H. (2025). Non-invasive detection of arsenic-induced changes in lentil seed activity using biospeckle optical coherence tomography. Opt. Continuum 4, 968–984. doi: 10.1364/optcon.555904

[B5] Iqbal M. M. Farooq M. A. Khan W. Ashraf U. Alfaghom A. T. Alamri S. (2025). Maize growth and physiological dynamics: Arsenic uptake modulation under combined abiotic stresses of salinity, boron and arsenic. Environ. Technol. Innovation 37, 103915. doi: 10.1016/j.eti.2024.103915 38826717

[B8] Karimi N. Nosrati B. Souri Z. Vaculík M. (2025). The dual role of sulfur and selenium in mitigating arsenic toxicity in Isatis cappadocica Desv.: A physiological, and ionomic analysis of stress responses. J. Soil Sci. Plant Nutr. 25, 8941–8950. doi: 10.1007/s42729-025-02704-5 30311153

[B10] Patel K. S. Pandey P. K. Martin-Ramos P. Corns W. T. Varol S. Bhattacharya P. . (2023). A review on arsenic in the environment: contamination, mobility, sources, and exposure. RSC Adv. 13, 8803–8821. doi: 10.1039/d3ra00789h 36936841 PMC10020839

[B11] Saifullah Dahlawi S. Naeem A. Iqbal M. Farooq M. A. Bibi S. . (2018). Opportunities and challenges in the use of mineral nutrition for minimizing arsenic toxicity and accumulation in rice: A critical review. Chemosphere 194, 171–188. doi: 10.1016/j.chemosphere.2017.11.149 29202269

[B12] Sinha D. Datta S. Mishra R. Agarwal P. Kumari T. Adeyemi S. B. . (2023). Negative impacts of arsenic on plants and mitigation strategies. Plants-Basel 12, 1815. doi: 10.3390/plants12091815 37176873 PMC10181087

[B13] Souri Z. Khanna K. Karimi N. Ahmad P. (2021). Silicon and plants: Current knowledge and future prospects. J. Plant Growth Regul. 40, 906–925. doi: 10.1007/s00344-020-10172-7 30311153

[B14] Souri Z. Sharifan H. de Oliveira L. M. Ngatia L. (2022). “ Arsenic removal by phytoremediation techniques,” in Arsenic in plants: uptake, consequences and remediation techniques (United States: John Wiley & Sons). doi: 10.1002/9781119791461.ch14

[B15] Su Q. T. Du Z. X. Huang X. Y. Hassan M. U. Altihani F. A. (2025). Managing arsenic pollution from soil-plant systems: Insights into the role of biochar. Plants-Basel 14, 1553. doi: 10.3390/plants14101553 40431117 PMC12115003

[B16] Vaculík M. Lukačová Z. Bokor B. Martinka M. Tripathi D. K. Lux A. (2020). Alleviation mechanisms of metal(loid) stress in plants by silicon: a review. J. Exp. Bot. 71, 6744–6757. doi: 10.1093/jxb/eraa288 32569367

